# EEG Signal Processing Techniques and Applications—2nd Edition

**DOI:** 10.3390/s25030805

**Published:** 2025-01-29

**Authors:** Hua-Liang Wei, Yuzhu Guo, Fei He, Yifan Zhao

**Affiliations:** 1 Department of Automatic Control and Systems Engineering, School of Electrical and Electronic Engineering, The University of Sheffield, Sheffield S1 3JD, UK; w.hualiang@sheffield.ac.uk; 2 School of Automation Science and Electrical Engineering, Beihang University, Beijing 100191, China; yuzhuguo@buaa.edu.cn; 3 Centre for Computational Science and Mathematical Modelling, Coventry University, Coventry CV1 2JH, UK; 4 Faculty of Engineering and Applied Sciences, Cranfield University, Cranfield MK43 0AL, UK

## 1. Introduction

Electroencephalography (EEG), as a well-established, non-invasive tool, has been successfully applied to a wide range of conditions due to its many evident advantages, such as economy, portability, easy operation, easy accessibility, and widespread availability in hospitals. EEG signals, with ultra-high time resolution, are vital in understanding brain functions. Traditionally, considerable attention in EEG signal processing and analysis has been paid to understanding brain activities from various perspectives, such as the detection and identification of abnormal frequencies in specific biological states, spatial–temporal and morphological characteristics of neurological disorder behaviours (e.g., paroxysmal or persistent discharges), the response of the brain nervous/neurological system to external stimuli, and the effects and responses to intermittent photic stimulation [1].

The past few years have seen rapid and significant advancement in signal processing, signal-based analysis, artificial intelligence (AI), machine learning (ML), and many other signal-based and data-driven techniques, propelling EEG signal processing into a new era with exciting progress in many areas, in order to meet growing demands and challenges in various real applications [2]. For example, some important nonlinear features of brain dynamics, which cannot be uncovered using traditional methods, may be revealed through analyzing associated EEG signals using state-of-the-art techniques and therefore facilitate the applications of EEG in various fields [3,4].

Recent years have witnessed an increasing number of EEG signal processing applications, aided by ML, AI, and other signal-based techniques in nearly all fields of science and engineering including neuroscience [5], clinical studies [6], brain–machine interfaces [7], cognitive science and psychology [8], human factors [9], and social interactions [10], to mention but a few. Methods and algorithms have been or are being developed to solve either the existing problems or emerging challenges faced by the world [11].

It is now the right time to delve further and deeper into investigations of EEG signal processing techniques. This Special Issue serves as a platform for the dissemination of the latest research results, findings, and trends in EEG signal processing and their applications, with particular attention to applications of machine learning and deep neural network methods. A total of 18 papers were collated as a part of this Special Issue and they can be roughly classified into six groups as follows:

- Brain–computer interfaces (Papers 1, 7, 11, and 16)
- Brain and neurological disorder detection and diagnosis (Papers 2, 3, 9, 12, 14, and 17)
- Cognitive and psychology studies (Papers 4, 10 and 15)
- Healthcare including mental health, pain identification, and depression diagnosis (Papers 5, 8, and 13)
- Brain functional connectivity (Paper 6)
- EEG artifact reduction and removal (Paper 18)

## 2. Overview of Contributions

In the following section, a short overview of each of the above six topics is provided, followed by a brief summary of each of the papers in the corresponding topic group.

### 2.1. EEG-Based Brain–Computer Interface

An EEG-based Brain–Computer Interface (BCI) is a system that uses EEG electrodes to measure brain activity and translate the associated signals into specific commands to drive external devices [12]. Initially, these applications were developed to assist patients, helping them regain normalcy in their lives. However, over time, BICs have also found significant use in non-medical domains, improving efficiency and collaboration among healthy individuals and aiding in personal development [12].

The authors of Paper 1 (Mwata-Velu et al.) proposed an embedded multi-task classifier based on motor imagery using the EEGNet toolbox (which is a compact Convolutional Network platform for EEG-based BCIs) and implemented the designed BCI system into a NVIDIA Jetson TX2 hardware platform. The performance of the proposed EEG-BCI system was tested on a public dataset, with the experimental results showing that the proposed system was suitable for online applications.

The authors of Paper 7 (Farabbi and Mainardi) presented a novel approach to enhancing Error Potential (ErrP) detection during single-trial (ST) stimulation tasks using conventional Convolutional Neural Networks (CNNs). The performance of the proposed approach was tested on an open-access EEG dataset, with the experimental results providing strong evidence that the proposed method was highly effective in improving ST-ErrP accuracy compared with several baseline methods.

The authors of Paper 11 (Khabti et al.) proposed a new Fusion Convolutional Neural Network with Attention blocks (FCNNA) model for optimal channel selection and multiclass Motor Imagery (MI) classification. The experimental results on a benchmark dataset (i.e., the BCI IV 2a dataset) showed that the proposed EEG-MI model outperformed the compared channel selection and classification methods.

The authors of Paper 16 (Dillen et al.) proposed an innovative control approach to assistive robotics by integrating BCI and eye-tracking techniques into a shared control system for a mobile augmented reality user interface. The system was designed to facilitate individuals with physical disabilities, particularly those with impaired motor function. While the research findings indicated that the shared BCI control system is effective for task completion and demonstrated the feasibility of the shared control strategy, the current efficiency of the BCI still requires further improvement for practical real-world applications.

### 2.2. Brain and Neurological Disorder Detection and Diagnosis

Brain and neurological disorders represent major global healthcare issues. Early detection of any disorder is crucial for curing patients or helping to prevent disease progression. Signal-based and data-driven modelling techniques, such as time–frequency analysis, information theory, machine learning, and artificial intelligence methods, are being increasingly used in brain and neurological disorder detection and diagnosis [13].

The authors of Paper 2 (Jurdana et al.) introduced a novel method for estimating instantaneous frequencies and group delays, which can be used to better detect seizures with both spike and oscillatory characteristics. The main advantage of the proposed method is that it makes use of Localised Rényi Entropies (LREs) to generate time–frequency information that better characterises the relevant signals.

The authors of Paper 3 (Vitório et al.) aimed to understand Parkinson’s disease (PD) by analysing the associated scalp EEG (sEEG) signals. The authors delve into investigating whether PD patients present distinct brain electrocortical activity during regular walking and during obstacle avoidance walking in comparison with healthy individuals. Experiments were carried out on 14 healthy older adults and 15 patients with PD. The research findings suggest that the PD patent EEG signals showed a greater proportion of low-frequency neuronal firing in brain areas related to motor commands and sensorimotor integration during walking.

The authors of Paper 9 (Vieira et al.) presented a feature dimensionality reduction method for epileptic seizure detection, aimed at reducing the number of channels required for classification and therefore making better use of the interpretability of machine learning models. The performance of the proposed method was tested on a publicly accessible dataset provided by the University of Beirut Medical Center. The proposed method showed an advantage in solving tasks with a relatively smaller number of channels, enabling the development of effective mobile applications for epileptic seizure detection.

The authors of Paper 12 (Wang et al.) proposed a feature extraction method by designing a multimodal dual-stream neural network model, constructed using convolution and Long Short-Term Memory (LSTM) neural networks. An advantage of the proposed method is that it can make use of several types of features in time and frequency domains, in addition to various signal differential features. According to the experimental results for experiments performed on several benchmark datasets, the proposed method outperformed comparable methods.

The authors of Paper 14 (Zhou et al.) designed a novel and interesting unsupervised approach for exploratory EEG analysis by defining low-dimensional prototypes in latent space, based on which EEG clustering and classification are performed. The proposed method was acquired by using Wavelet transform, a Generative Adversarial Network (GAN), and an extended Stein Latent Optimisation (SLO) scheme for the GAN. The proposed approach, W-SLOGAN, showed promising performance for diagnosing epilepsy subtypes and classifying multiple labelled EEG data.

The authors of Paper 17 (Aziz et al.) introduced an innovative automated approach for detecting Schizophrenia based on EEG signals. The approach was developed by using a fast independent component analysis method to remove artefacts from raw EEG data first and then using a novel Automated Log Energy-based Empirical Wavelet Reconstruction (ALEEWR) method to reconstruct decomposed signals to obtain relevant EEG signatures. The results of experiments performed on a benchmark dataset showed that the proposed approach appeared to achieve exceptionally excellent performance for Schizophrenia detection compared to many existing methods.

### 2.3. EEG for Cognitive and Psychology Studies

In addition to its application in studying brain and neurological disorders as mentioned above, EEG has been increasingly applied to different areas of cognitive and psychology research, such as cognitive load, attention, memory, and emotional processing [14,15]. One important application is neurofeedback training, where individuals learn to regulate their brain activity, showing promise in treating conditions such as Attention-Deficit/Hyperactivity Disorder (ADHD) and anxiety [16]. EEG is also used in conjunction with other neuroimaging techniques, such as fMRI and MEG, to provide a more comprehensive and better understanding of brain dynamics [17].

The authors of Paper 4 (Wang et al.) presented a feasibility study on investigating drivers’ brain activity patterns by performing simulations of various levels of cognitive load based on four designed driving tasks. The authors used deep neural networks and four Support Vector Machines (SVMs) to classify EEG signals measured to differentiate driving conditions. The research results and findings show potential in improving the performance of the human–machine interface of vehicles and thus help to improve safety.

The authors of Paper 10 (Phukhachee et al.) introduced a new method to identify the cognitive motivation effect with a reduced number of EEG electrodes. The authors hypothesised that the temporal relationship of brain activities between attention- and memorisation-related areas could aid in identifying the effect of motivation on remembering the associated stimulus, with the number of electrodes reduced to two (i.e., the FCz and P3 electrodes). They proposed a method based on the temporal association rule mining (TARM) concept to identify the motivation effect from the temporal relationship of brain activities between attention and memorisation areas while the participants are being motivated. The proposed approach was implemented using an SVM, whose hyper-parameters were obtained by using the Artificial Bee Colony (ABC) algorithm. The results of experiments on a benchmark dataset provide valuable support for the original hypothesis.

The authors of Paper 15 (Ji et al.) carried out investigations on understanding and detecting pilots’ psychological workload during turning phases using EEG signals collected from pilots during left and right turns in simulated flight scenarios. The analysis includes the changes in EEG signals, variations in EEG power, and the correlations between EEG power and turning maneuvers. The results given by the designed SVM classifier showed that significant changes occurred in the energy ratio of beta waves and Shannon entropy during left and right turns compared to the cruising phase. The research findings are potentially useful for flight training and enhancing flight safety.

### 2.4. Healthcare—Mental Health, Pain Identification, and Depression Diagnosis

EEG represents a powerful tool in healthcare, particularly for mental health, pain identification, and depression diagnosis. In mental health, EEG is used to diagnose and monitor psychiatric and neuropsychiatric disorders [18,19]. It helps in identifying abnormalities in brain activity that may be associated with conditions such as epilepsy, which often coexists with psychiatric disorders [19]. EEG can also be used to study and modify local cerebral disorders related to abnormal behaviour [18]. In pain identification, EEG-based pain identification involves analyzing brain signals to detect and quantify pain [20,21]. In depression diagnosis, EEG is used in diagnosing depression by detecting specific physiological changes in the brain [22]. Advanced machine learning and deep learning techniques have been applied to EEG data to improve diagnostic accuracy [23]. These methods involve the analysis of neural oscillations and asymmetries in brain activity to identify depression more precisely.

The authors of Paper 5 (Xu et al.) proposed a framework for depressive disorder (DD) recognition based on six frontal-channel EEG data sources. Two deep learning models, a multi-resolution CNN (MRCNN) combined with LSTM and an MRCNN combined with residual squeeze and excitation (RSE), were built to extract features and classify EEG signals. The results of experiments performed on a publicly available dataset with 128 EEG channels showed that the proposed approach effectively diagnosed depressive disorder.

The authors of Paper 8 (Alreshidi et al.) focused on predicting pilot mental states using EEG data. They developed an interpretable model to detect four mental states, namely, channelised attention, diverted attention, startle/surprise, and normal state. The SHapley Additive exPlanations (SHAP) values were used to identify the top 10 most influential features for each mental state. The work represents a significant advancement in the field of EEG-based pilot mental state detection.

The authors of Paper 13 (Segning et al.) presented a pilot study on the detection and evaluation of the magnitude of chronic pain. They introduced a scale-independent measure, referred to as the coefficient of variation of the upper envelope (CVUE), to characterise the associated EEG signals and used the measure to compare the degree of variation from one time-series to another. Experiments were carried out on three groups of volunteers, involving 41 participants with different types and different levels of chronic pain. The experimental results showed that the proposed method can effectively quantify pain in a population living with chronic pain.

### 2.5. Brain Functional Connectivity

Functional connectivity (FC) is a concept in neuroscience that is concerned with the temporal dependency of neuronal activation patterns in different brain regions, reflecting the statistical dependencies between these areas. Essentially, this concept is concerned with understanding how different parts of the brain communicate with one another over time. The main statistical measures used in FC include correlation, covariance, spectral coherence, and phase locking. Correlation measures the strength and direction of the linear relationship between two variables. Covariance indicates the extent to which two variables change together. Spectral coherence assesses the consistency of the phase relationship between two signals across different frequencies. Phase locking measures phase synchronisation between two signals. These dependencies can be highly time-dependent, fluctuating on multiple time scales from milliseconds to seconds, reflecting the dynamic nature of brain activity. Functional connectivity has important applications in both research and clinical settings. It can be used to understand normal brain function, identify biomarkers for neurological and psychiatric disorders, and even guide interventions and treatments [24].

The authors of Paper 6 (Siviero et al.) applied a Bayesian estimation approach to estimate Transcranial Magnetic Stimulation (TMS)-evoked potentials (TEPs) from EEG data; such an approach has not been investigated in the context of transcranial magnetic stimulation combined with electroencephalography (TMS–EEG). The authors designed a self-tuning optimised Kalman (STOK) filter in conjunction with the information partial directed coherence (iPDC) measure to capture the rapid dynamics of information flow patterns, based on which time-varying connectivity matrices were derived. Graph analysis was then conducted to assess key network properties, offering a better understanding of how visual information is propagated across brain networks.

### 2.6. EEG Artifact Reduction and Removal

Artifacts are always undesired in EEG modelling and analysis as they distort the measurements of the signals of interest [25]. EEG signals can be compromised to some degree in either the time or frequency domain or both by artifacts stemming from internal or external sources [26]. There are a few major challenges in EEG artifact reduction and removal in many real applications, such as the requirement of calibration and effective evaluation criteria, the lack of EEG artifact benchmarks, and the existence of diversified artifacts.

The authors of Paper 18 (Hazarika et al.) proposed a novel approach that employed the artifact subspace reconstruction (ASR) algorithm to remove artifacts from single-channel EEG data. They introduced an embedded ASR (E-ASR) method to improve the efficiency of artifact removal. The proposed method was tested on a self-created, semi-simulated dataset. The experimental results showed the excellent overall performance of the proposed approach for handling single-channel EEG data.

## References

[1] Sanei S., Chambers J.A. (2021). EEG Signal Processing and Machine Learning.

[2] Hosseini M.P., Hosseini A., Ahi K. (2020). A Review on machine learning for EEG signal processing in bioengineering. IEEE Rev. Biomed. Eng..

[3] Gu Y., Yang Y., Dewald J.P.A., van der Helm F.C.T., Schouten A.C., Wei H.-L. (2021). Nonlinear modeling of cortical responses to mechanical wrist perturbations using the NARMAX method. IEEE Trans. Biomed. Eng..

[4] Cao J., Zhao Y., Shan X., Wei H., Guo Y., Chen L., Erkoyuncu J.A., Sarrigiannis P.G. (2022). Brain functional and effective connectivity based on electroencephalography recordings: A review. Hum. Brain Mapp..

[5] Ienca M., Ignatiadis K. (2020). Artificial Intelligence in Clinical Neuroscience: Methodological and Ethical Challenges. Neuroscience.

[6] Tveit J., Aurlien H., Plis S., Calhoun V.D., Tatum W.O., Schomer D.L., Arntsen V., Cox F., Fahoum F., Gallentine W.B. (2023). Automated Interpretation of Clinical Electroencephalograms Using Artificial Intelligence. JAMA Neurol..

[7] Wang X., Hersche M., Magno M., Benini L. (2024). Mi-bminet: An efficient convolutional neural network for motor imagery brain–machine interfaces with eeg channel selection. IEEE Sens. J..

[8] Gong X., Li X. (2023). Human–robot interactive communication and cognitive psychology intelligent decision system based on artificial intelligence—Case study. Int. J. Humanoid Robot..

[9] Chignell M., Wang L., Zare A., Li J. (2023). The evolution of HCI and human factors: Integrating human and artificial intelligence. ACM Trans. Comput.-Hum. Interact..

[10] Bolotta S., Dumas G. (2022). Social neuro AI: Social interaction as the “dark matter” of AI. Front. Comput. Sci..

[11] Ein Shoka A.A., Dessouky M.M., El-Sayed A., Hemdan E.E.D. (2023). EEG seizure detection: Concepts, techniques, challenges, and future trends. Multimed. Tools Appl..

[12] Lotte F., Bougrain L., Cichocki A., Clerc M., Congedo M., Rakotomamonjy A., Yger F. (2018). A review of classification algorithms for EEG-based brain–computer interfaces: A 10 year update. J. Neural Eng..

[13] Acharya U.R., Oh S.L., Hagiwara Y., Tan J.H., Adeli H. (2018). Deep convolutional neural network for the automated detection and diagnosis of seizure using EEG signals. Comput. Biol. Med..

[14] Allen J.J.B., Keune P.M., Schönenberg M., Nusslock R. (2018). Frontal EEG alpha asymmetry and emotion: From neural underpinnings and methodological considerations to psychopathology and social cognition. Psychophysiology.

[15] Babayan A., Erbey M., Kumral D., Reinelt J.D., Reiter A.M., Röbbig J., Schaare H.L., Uhlig M., Anwander A., Bazin P.L. (2019). A mind-brain-body dataset of MRI, EEG, cognition, emotion, and peripheral physiology in young and old adults. Sci. Data.

[16] Markovska-Simoska S., Pop-Jordanova N. (2017). Quantitative EEG in Children and Adults with Attention Deficit Hyperactivity Disorder: Comparison of Absolute and Relative Power Spectra and Theta/Beta Ratio. Clin. EEG Neurosci..

[17] Garcés P., Pereda E., Hernández-Tamames J.A., Del-Pozo F., Maestú F., Pineda-Pardo J. (2015). Ángel Multimodal description of whole brain connectivity: A comparison of resting state MEG, fMRI, and DWI. Hum. Brain Mapp..

[18] Badrakalimuthu V.R., Swamiraju R., de Waa H. (2011). Is EEG a useful test in adult psychiatry?. Adv. Psychiatr. Treat..

[19] Sand T., Bjørk M.H., Vaaler A.E. (2013). Is EEG a useful test in adult psychiatry?. Tidsskr. Nor. Legeforen..

[20] Prichep L.S., Shah J., Merkin H., Hiesiger E.M. (2018). Exploration of the pathophysiology of chronic pain using quantitative EEG source localization. Clin. EEG Neurosci..

[21] Sun G., Wen Z., Ok D., Doan L., Wang J., Chen Z. (2021). Detecting acute pain signals from human EEG. J. Neurosci. Methods.

[22] Steiger A., Kimura M. (2010). Wake and sleep EEG provide biomarkers in depression. J. Psychiatry Res..

[23] Khosla A., Khandnor P., Chand T. (2022). Automated diagnosis of depression from EEG signals using traditional and deep-learning approaches: A comparative analysis. Biocybern. Biomed. Eng..

[24] Lang E.W., Tomé A.M., Keck I.R., Górriz-Sáez J.M., Puntonet C.G. (2012). Brain connectivity analysis: A short survey. Comput. Intell. Neurosci..

[25] Urigüen J.A., Garciazapirain B. (2015). EEG artifact removal—State-of-the-art and guidelines. J. Neural Eng..

[26] Islam M.K., Rastegarnia A., Yang Z. (2016). Methods for Artifact Detection and Removal from Scalp EEG: A Review. Clin. Neurophysiol..

